# Infant preference for specific phonetic cue relations in the contrast between voiced and voiceless stops

**DOI:** 10.1111/infa.12630

**Published:** 2024-11-01

**Authors:** Marc Hullebus, Adamantios Gafos, Natalie Boll‐Avetisyan, Alan Langus, Tom Fritzsche, Barbara Höhle

**Affiliations:** ^1^ Department of Linguistics University of Potsdam Potsdam Brandenburg Germany; ^2^ Haskins Laboratories Inc New Haven Connecticut USA

## Abstract

Acoustic variability in the speech input has been shown, in certain contexts, to be beneficial during infants' acquisition of sound contrasts. One approach attributes this result to the potential of variability to make the stability of individual cues visible. Another approach suggests that, instead of highlighting individual cues, variability uncovers stable relations between cues that signal a sound contrast. Here, we investigate the relation between Voice Onset Time and the onset of F1 formant frequency, two cues that subserve the voicing contrast in German. First, we verified that German‐speaking adults' use of VOT to categorize voiced and voiceless stops is dependent on the value of the F1 onset frequency, in the specific form of a so‐called trading relation. Next, we tested whether 6‐month‐old German learning infants exhibit differential sensitivity to stimulus continua in which the cues varied to an equal extent, but either adhered to the trading relation established in the adult experiment or adhered to a reversed relation. Our results present evidence that infants prefer listening to speech in which phonetic cues conform to certain cue trading relations over cue relations that are reversed.

## 1 INTRODUCTION

A crucial aspect of phonological development is that typically developing infants are highly efficient in detecting the acoustic‐phonetic parameters that express phonological contrasts in their ambient language. This capacity is remarkable given the rampant variability that characterizes speech both within and across speakers. For instance, contrasts between vowels (e.g., /i/ vs. /u/) are predominantly cued by the first three formant frequencies, the spectral maxima corresponding to the resonant frequencies of the vocal tract. It is a well‐documented fact that different speakers uttering phonologically identical vowels do not produce the same formant values (Peterson & Barney, 1952). Variability is similarly rampant for consonants. In many languages, a phonetic cue distinguishing phonologically voiced versus voiceless stop consonants (e.g., /d/ vs. /t/) in syllable‐initial position is Voice Onset Time (VOT), quantified by the duration between the onset of the burst created by the release of the oral occlusion of the consonant and the onset of the vocal fold vibration for the subsequent vowel (Lisker & Abramson, 1967). This cue has also been shown to display extensive variability both across and within speakers (e.g., for English, see Allen et al., 2003; for German, see Hullebus et al., 2018). Less emphasized in the context of acquisition is that the expression of phonological contrasts relies on multiple phonetic cues, usually combinations of spectral (as in formant frequencies) and durational (as in VOT) properties of the signal (Repp, 1982). More crucially, for current purposes, these cues interact with one another in perception. Thus, in languages like American English and Standard German, VOT is one (and in the syllable‐initial context after a pause, perhaps the most) important cue for signaling the distinction between voiced and voiceless stop consonants. Yet, other cues expressed in spectral dimensions, such as the frequency at the onset of the first formant or F1, have been shown to be crucial in perceiving whether a consonant is voiced or not (Jiang et al., 2006; Summerfield & Haggard, 1977). More specifically, in the perception of voicing in English, the two cues of VOT and F1 onset frequency enter into a so‐called trading relation (Repp, 1982): as F1 onset frequency increases, VOT must decrease for the perceptual identity of a voiced unaspirated stop to be maintained. Conversely, as F1 onset frequency decreases, VOT must increase for the perceptual identity of a voiceless aspirated stop to be maintained.

Recently, trading relations have come to the fore in the context of the broader aim to better understand the role of variability in language acquisition. Both in language development and other domains of cognition, there is broad evidence for the beneficial role of variability on category formation and generalization (Posner & Keele, 1968; Quinn & Bhatt, 2010). Variability in the speech signal, more specifically, has been shown to aid learning of phonological categories (Bortfeld & Morgan, 2010; Höhle et al., 2020; Quam et al., 2017; Rost & McMurray, 2009, 2010). The reasons for this are not clear. One view, expressed in Rost and McMurray (2010), is that variability helps learners home in on the right cues for phonological contrasts by tracking the extent of variability in individual cue candidates. In this view, relatively stable single cues are favored and variability or ‘noise’ in the speech signal is beneficial because it serves to highlight their stability. An alternative view, expressed in Höhle et al. (2020), holds that variability is beneficial not because it highlights the presumed invariant or relatively stable cues, but because it brings out relational properties among relevant cues. This view does not rest on the existence of some relatively stable—in the sense of invariant—cue but rather on the presence of stable relations among cues (where the individual cues themselves may very well vary). The plausibility of this alternative in the context of acquisition depends on evidence that infants are sensitive to relations between cues. It is the main aim of the present study to contribute such evidence.

While infants have been shown to be sensitive to subphonemic differences in single cues such as VOT at the age of at least 8 months (Lasky et al., 1975; Maye et al., 2002; McMurray & Aslin, 2005) and even 3 months (Miller & Eimas, 1996), as well as continuous formant cues at 2 months (Swoboda et al., 1976), much less is known about infants' sensitivity to relations between cues. Some studies from the 1980s indicate that at least certain cue relations are perceptually relevant early in language acquisition. In one of these studies, Miller and Eimas (1983) examined how 3‐ to 4‐month‐olds discriminated synthesized /da/‐/ta/ syllable pairs varying in VOT and F1 transition duration. The duration of the F1 transition from vowel onset to vowel midpoint is related to F1 onset frequency, the parameter we also manipulate in our study, as a longer transition usually implies a lower F1 onset frequency (Jiang et al., 2006; Lisker, 1975). Thus, the two cues trade for one another: when the F1 transition duration is short, implying a higher F1 onset frequency, then a shorter VOT is sufficient to signal a voiceless stop. In contrast, when the F1 transition is long, implying a lower F1 onset frequency, then a longer VOT is required to signal a voiceless stop. In attestation of this relation, Miller and Eimas (1983) demonstrated that infants perceived a stop with the same VOT (30 ms) as voiced when F1 transition duration was long (85 ms), but as voiceless when F1 transition duration was short (25 ms).

In a subsequent study, Eimas (1985) assessed another trading relation, that between F1 onset frequency and silence duration in the perception of a stop versus fricative contrast in the *say‐stay* word pair by 4‐month‐olds. An F1 rising from a low(er) onset frequency indicates the release of a constriction (i.e., the presence of stop consonant, the [t] in *stay*) just as a long(er) silence duration does (silence being the prototypical cue for a stop). Here too, the two cues trade for one another: less silence duration is required to signal a stop when the spectral cue has more extreme values (either low F1 onset frequency or longer F1 transition duration; see Summerfield & Haggard, 1977; Bailey & Summerfield, 1980 for findings on adults). Eimas (1985) demonstrated that 4‐month‐olds discriminated between the two words *say* and *stay* when the F1 frequency and silence duration cues conformed to this trading relation. That is, a lower F1 onset combined with a longer silence duration corresponded to the presence of the stop, while a higher F1 onset combined with a shorter silence duration corresponded to the absence of the stop. In contrast, the infants did not discriminate the stimuli when the two cues did not conform to the trading relation, that is, a lower F1 onset combined with a shorter silence duration and a higher F1 onset combined with a longer silence duration. Moreover, some evidence exists that sensitivity to this relation develops with age. Using the same contrast but with a more densely sampled F1 continuum, Morrongiello et al. (1984) found that the F1 cue did not affect the category boundary between *say* and *stay* as strongly for 5‐year‐olds as for adults (see also Simon & Fourcin, 1978, for results using the VOT and F1 transition duration trading relation from children learning British English).

The current study builds on these earlier findings by comparing infants' responses to stimuli with cues that conform or do not conform to the trading relation between VOT and F1 onset frequency. Recall that Miller and Eimas (1983) assessed the relation between VOT and F1 transition duration, which is related but not identical to the relation we assess here. There is, furthermore, evidence that F1 transition duration is not as strong as F1 onset frequency in influencing the perception of voicing (Jiang et al., 2006; Lisker, 1975). Hence, as far as we know, despite robust evidence for its presence in adult perception, whether infants are sensitive to the VOT‐F1 onset frequency relation has not been addressed before.

As a prerequisite in assessing the preference for stimuli adhering to cue relations in infants, we first intend to establish the presence of the trading relation between VOT and F1 onset frequency in German adult listeners' perception of the voicing contrast in Experiment 1. While previous work on English and Spanish (Benkí, 2005; Jiang et al., 2006; Lisker, 1975) suggests that this relation should also be present in other languages, we know of no prior work which provides evidence for this expectation in German. Having established the presence of the trading relation in German, we then turn to Experiment 2, addressing whether a difference in preference for stimuli adhering to the cue trading relation over a reversed relation is present in 6‐month‐old German learning infants.

## 2 EXPERIMENT 1

### 2.1 Methods

#### 2.1.1 Participants

Eighteen monolingual German speakers (14 female, 4 male) participated in the experiment. All participants were students at the University of Potsdam, Germany. They received either course credit or monetary compensation. None of the participants reported diminished hearing capacity or language disorders. All had normal or corrected‐to‐normal vision. The participants' average age was 21.4 years (SD = 2.99), ranging between 17.7 and 27.2 years. The target sample size was informed by results of a pilot study with synthesized stimuli and a comparable prior experiment (Benkì, 2001). Data collection proceeded until the target size was reached. The experiment was conducted according to guidelines laid down in the Declaration of Helsinki, with written informed consent obtained from each participant before data collection. All procedures involving human subjects in this study were approved by the Ethics Committee at the University of Potsdam.

#### 2.1.2 Stimuli

The stimuli for the adult participants in Experiment 1, as well for the infants in Experiment 2, were based on recordings of a female native speaker of Northern German. The model speaker was instructed to produce nonce words in a mildly infant‐directed manner so that the stimuli would be suitable for use in an infant experiment. The stimuli were recorded in a sound‐attenuated booth using an Audio Technica AT4033a large diaphragm condenser microphone with an M‐Audio interface set with peaks at approximately −10 dB below the clipping point.

All nonce words had a [ˈCaːvə] form, where ‘C’ denotes an alveolar /d/ or /t/ stop consonant followed by [aːvə], that is, a low back vowel in the first stressed syllable and a labiodental voiced fricative followed by a schwa in the second, unstressed syllable. Being a common pattern in German, the disyllabic schwa‐final structure is a plausible German word candidate such that German‐learning infants would readily accept the nonce as native‐sounding. The low back vowel /a:/ was selected because the F1 transition in alveolar stops is particularly prominent in back vowels. From the set of infant‐directed [ˈCaːvə] recordings, we selected one voiceless aspirated and one voiced unaspirated token to serve as the endpoints of the target VOT continuum. The selection criteria were that the F1 frequency in the steady portion of both stimuli was approximately equal (around 1000 Hz), while the F1 onset frequency of the voiced stop was to be comparatively low (600 Hz) to allow for a wide continuum. With regards to VOT, a syllable‐initial stop in Standard German is perceived as voiced, otherwise known as lenis, with a relatively short VOT of around 10–15 ms, while a longer VOT of around 40–50 ms is perceived as voiceless or fortis (Jessen, 1999; Lein et al., 2016). Thus, we chose a voiced stimulus with a VOT of 5 ms and a voiceless counterpart with a VOT of 40 ms. From these endpoints, continua were created in which the two cues could covary so that the relation between the cues is exemplified across a range of values spanning the voicing category boundaries.

Given two naturally recorded speech signals that serve as endpoints, it is possible to create a continuum between them using Tandem‐STRAIGHT (Kawahara et al., 2008), a MATLAB‐based environment for analyzing and manipulating speech. Using this software, the pitch contour, duration and spectral characteristics are controlled not by splicing or averaging but by so‐called ‘morphing’: The user defines time and frequency anchors within each of the two endpoint stimuli, so that the temporal and spectral characteristics of the stimuli on the generated continuum can shift between the anchors rather than merely being averaged together, which reduces noise and artifacts. An eight‐step VOT continuum between 5 and 40 ms in increments of 5 ms was generated by morphing between voiced and voiceless stops in the aperiodic portion of the signal. The remaining part of the stimulus was identical for all stimuli as a midway ‘morph’ between the two endpoints, such that no cues in the remaining part of the signal would point to either voiced or voiceless category.

Since Tandem‐STRAIGHT does not readily allow for a precise manipulation of formant transitions, we used Praat (Boersma & Weenink, 2015) to perform formant manipulations. A spectral slice was made between the lower (600 Hz) and upper (1000 Hz) F1 frequency range for the duration of the F1 transition (40 ms). The F1 formant transitions were subsequently manipulated to start at 6 different onset frequencies between the upper and lower bounds using linear predictive coding (LPC) decomposition, while the transition duration was kept constant. The unaltered spectrum above 1500 Hz was subsequently spliced onto the manipulated spectral slice to retain naturalistic qualities in the high‐frequency parts of the stimuli. This process resulted in 6 F1 transition steps for each of the 8 VOT steps for a total of 48 distinct stimuli in the two‐dimensional VOT‐F1 continuum. Finally, the stimuli were all normalized to 71 dB‐SPL. An overview of the endpoints and final VOT‐F1 continuum is illustrated in Figure 2.

#### 2.1.3 Procedure

The experiment was a two‐alternative forced choice task in which participants had to judge if an auditory stimulus started with either ‘da’ or ‘ta’ by pressing a corresponding key on a laptop keyboard. The stimuli and responses were presented and collected using OpenSesame (Mathôt et al., 2012). Each of the 48 stimuli in the VOT‐F1 continuum was repeated five times in shuffled order, totaling 240 trials. Participants wore Shure SRH440 headphones and were seated in a sound‐attenuated booth. While the audio played, a fixation cross was visible on a gray background on the laptop screen. This was followed by a screen showing both “da” and “ta” alternatives placed adjacent in the middle of the screen on the side that corresponded to the key press: *c* to the left above the space bar and *m* to the right. The response location was counterbalanced across participants, so that for one half of the participants, the voiced stop corresponded to the left key and for the other half the voiced stop corresponded to the right key. If the participants needed a short break during the experiment, they were able to interrupt the experiment at will; two participants chose to do so. The total duration of the experiment was approximately 15 min.

### 2.2 Results and discussion

The anonymized data can be found on OSF: DOI 10.17605/OSF.IO/C7W8Q (https://osf.io/C7W8Q). Button presses were converted to a percentage of voiceless /ta/ responses of total responses. Trials for which participants took longer than 3000 ms after stimulus onset to respond were not recorded (as in Benkí, 2001). Trials with response times less than 100 ms were excluded from the analysis, as this can be considered a minimum time necessary to generate a physiological response (Luce, 1986). This resulted in an exclusion rate of 2.3% of total responses. The mean response time was 664 ms.

**FIGURE 1:**
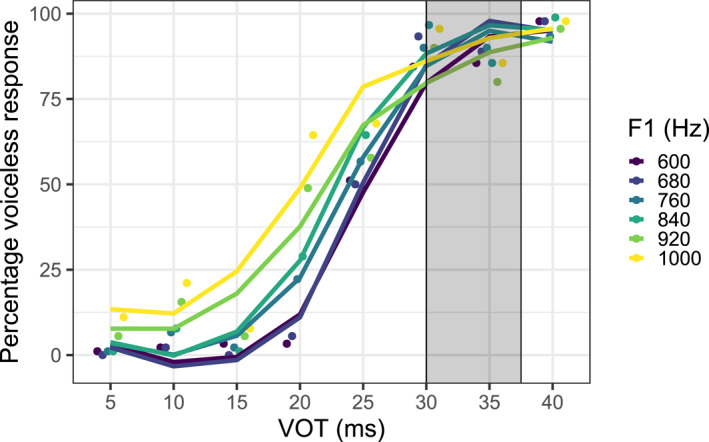
Percentage of voiceless responses (*y*‐axis) to stimuli varying in VOT (ms, *x*‐axis) and F1 (Hz, colors, dark to light for low to high F1). Regression lines are fit over responses (dots). Overlayed in gray are results of a Johnson‐Neyman test showing non‐significant regions of the interaction between VOT and F1.

Figure 1 displays the percentage of voiceless responses as a function of VOT on the *x*‐axis and of F1 by the differently colored lines. Dots represent the proportion of voiceless responses per stimulus type averaged across participants, while the lines are logistic regressions fits of the pooled data. Lighter colored lines represent higher F1 onset frequencies. As expected for German stops, the percentage of voiceless responses overall increases as VOT lengthens. Crucially, as F1 onset frequency increases, the number of voiceless responses rise, seen by the decreasing slopes as F1 onset frequency increases and the concomitantly less categorical discrimination curve.^1^

A logistic mixed‐effects regression model was fit using the *glmer* function in the R package lme4 (Bates et al., 2015). The proportion of voiceless responses was predicted by continuous fixed effects for VOT and F1, an interaction for VOT and F1 and random intercepts for subjects. Both predictors were scaled by subtracting the mean and dividing by the standard deviation due to their magnitude differences. A summary of the model is provided in Appendix A Table A1. Model fits indicated that both main effects for VOT (*β* = 2.89, *p* < 0.001, *z* = 33.22) and F1 (*β* = 0.56, *p* < 0.001, *z* = 11.05) were significant, as well as the interaction between VOT and F1 (*β* = −0.61, *p* < 0.001, *z* = −7.92). To analyze the interaction, a post‐hoc Johnson‐Neyman test was performed. For all the stimuli with identical VOT, the test calculates whether F1 onset frequency significantly affects the proportion of voiceless responses. This procedure revealed that higher F1 values, as expected, significantly increased the number of voiceless categorizations of stimuli for VOT steps up to 30 ms, as illustrated in Figure 1. Above 30 ms, the effect of F1 is not significant and for the last step of 40 ms, the test estimated a negative effect. This may be explained by a decrease in the slope of the effect for the highest F1 which appears to be present at VOTs over 30 ms: Modeling the subset of responses above 30 ms by individual F1 steps showed that while higher F1 overall results in a higher proportion of voiceless responses, the effect of the highest F1 steps decreases. The combined effect of both cues at the upper limits may have also been interpreted by listeners as too extreme and less prototypical, although differences near ceiling are small and difficult to interpret.

In summary, Experiment 1 sought to confirm the presence of a VOT‐F1 cue trading relation in German‐speaking adults, which, although expected based on the results in other languages, has not been established before. The findings reveal similar results to those of Benkí (2005) for English and Spanish: while VOT is as expected a dominant predictor of categorizing a stop as voiced or voiceless (with longer VOT values producing more voiceless categorizations), its effect on listeners' responses is significantly modulated by F1 onset frequency. This is most readily observed by the pattern of responses in the lower half of the VOT continuum in Figure 1. The higher the F1 onset frequency is, the more likely listeners are to categorize the stop as voiceless, as evidenced by the larger spread in the voiceless responses as a function of F1. At higher VOT steps (above 30 ms), however, as the proportion of voiceless responses approaches the ceiling, the effect of F1 on categorization has diminishing returns. This is reasonable, since the longest VOTs in the continuum provide overwhelming evidence for a voiceless stop, such that changes in F1 frequency minimally influence categorization. Moreover, our results demonstrate that the perceptual responses to manipulated stimuli, when spoken in a mildly infant‐directed manner, reveal similar cue trading relations as synthesized stimuli in previous studies. Since the F1 range in this experiment was modeled after the productions of a female German reference speaker and was also higher than in previous studies, the results show that the relation holds for the higher F1 frequency range used in the current experiment as well. With these results at hand, the following experiment turns to assess whether infant listeners prefer stimuli selected in accordance with the trading relation to stimuli where this relation is reversed.

## 3 EXPERIMENT 2

### 3.1 Methods

Having established that adult speakers of German show the expected perceptual trading relation between VOT and F1 onset frequency, we now turn to the experiment with infants. Using the head‐turn preference procedure as a standard measure for infants' preference for one type of stimuli over another, we exposed 6‐month‐old German learning infants to stimuli which either adhered to the relation or did not. We chose the age of 6 months since infants of that age have had experience in their native language but the native language‐specific constraints on consonant categorization before the age of 9 months remain flexible (e.g., Best, 1993; Jusczyk et al., 1999) and become mostly native‐like by 12 months (Werker & Tees, 1984). Departing from previous studies, infants were not tested with single exemplars of stimuli but with two sets of stimuli which represented continua between the stimuli conforming to the trading relation or a reversed relation. If infants are sensitive to the trading relation, we expect significant differences in listening time between the conforming and the reversed condition. We predict this difference to be a familiarity effect with infants listening longer to the conforming compared to the reversed condition.

#### 3.1.1 Participants

Twenty‐four 6‐month‐old monolingual German‐learning infants participated in the experiment. Caregivers reported no signs of developmental or hearing impairments, and none of the infants were born preterm. Recruitment was accomplished via the University of Potsdam's BabyLAB database. For their participation, caregivers received monetary compensation as well as a certificate of attendance. A further six infants were tested but excluded for not completing the experiment (4) or technical difficulties (2). The target sample size was determined to be a multiple of the counterbalanced list versions and to fall within the range of previous studies with comparable design (Höhle et al., 2009). Data collection continued until the target sample size was reached. The present study was conducted according to guidelines laid down in the Declaration of Helsinki, with written informed consent obtained from a parent or guardian for each child before any assessment or data collection. All procedures involving human subjects in this study were approved by the Ethics Committee at the University of Potsdam.

#### 3.1.2 Stimuli

**FIGURE 2:**
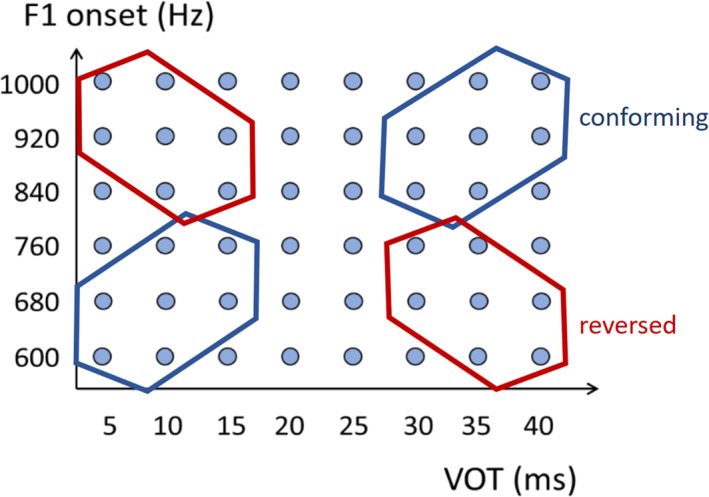
Individual stimuli for both experiments varying in VOT (ms, *x*‐axis) and F1 (Hz, *y*‐axis) represented as a matrix. The overlayed hexagons illustrate the selection of stimuli for Experiment 2: the dots in blue hexagons represent stimuli in the condition conforming to the VOT‐F1 trading relation, while the stimuli in the red hexagon represent the reversed relation.

Instead of densely sampling the complete VOT‐F1 space as was done in the adult experiment, we chose stimuli that either conform or do not conform to the trading relation at issue. Two groups of 14 stimuli for the head‐turn preference task were taken from the VOT‐F1 continua generated in Experiment 1, so that each trial would include some variability without direct repetitions of the same token. The groups of stimuli were selected based on the relation between the VOT and F1 cues. One group of the /Ca:/‐initial non‐words instantiated the *conforming* condition. Stimuli in this condition conformed to the earlier documented cue trading relation: higher VOT values were accompanied by higher F1 values and lower VOT values were accompanied by lower F1 values. A second group of stimuli instantiated a *reversed* relation between these two cues. Stimuli in this condition had lower VOT values co‐occurring with higher F1 values and vice versa. In Figure 2, the conforming condition stimuli are enclosed in a blue hexagon, while the reversed stimuli are enclosed in a red hexagon. Note that the stimuli in the ambiguous VOT range of 20 and 25 ms were not presented during the experiment. These ‘intermediate’ stimuli exhibit VOT‐F1 values that would fall within the range of both conditions.

#### 3.1.3 Procedure

The experiment used the head‐turn preference procedure (HPP; Hirsh‐Pasek et al., 1987) as in Kemler Nelson et al. (1995). Participants sat on their caregiver's lap in a three‐sided booth with lightbulbs as visual cues on each side. A green flashing light in front of the infants served to capture the infants' attention and (re‐)center their gaze between trials. Loudspeakers were placed on the left and right outside of the booth to play the auditory stimulus strings. Positioned in the center of the booth was a video camera, which allowed an experimenter to observe whether infants turned their head toward the visual cue. As the experimenter was seated in an adjacent room, the manual coding of the looking behavior was unaffected by the auditory stimuli. Before data analysis, the timestamps of live coding were compared to and, if necessary, corrected using the timestamps in the video recordings. After two warm‐up trials with music, infants heard stimulus strings from the VOT‐F1 continuum in two conditions: VOT and F1 in a relation conforming to the trading relation versus the reversed relation. For each trial, all instances from one corner in the stimulus matrix in Figure 2 were combined in random order (including one repetition), resulting in a sound file consisting of 14 tokens exhibiting variability in the VOT and F1 cues. The onsets of the tokens within each sound file were separated by 1.5 s, thereby setting the maximum trial length to 21 s. The trial duration was infant controlled: Following the standard procedure (Kemler Nelson et al., 1995), a trial was terminated if a child did not look for two consecutive seconds. There was a total of 16 trials per participant—four trials for each corner from the stimulus matrix, which were presented in pairs contrasting in cue relation condition. Which condition was presented first was counterbalanced across participants. The total experiment duration was approximately 5 min. Looking times were defined as the total duration of looks to the side of the booth where the trial is played. The prediction was that longer looking times should accompany the playback of stimuli exhibiting the conforming cue relation compared to those with a reversed cue relation.

### 3.2 Results and discussion

**FIGURE 3:**
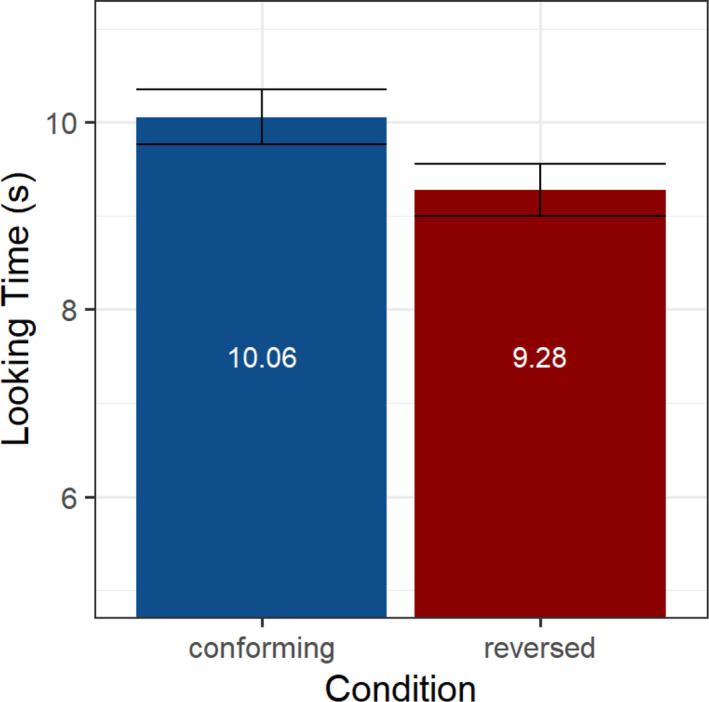
Barplot with looking times (s, *y*‐axis) during stimuli that conformed (left, blue) to the trading relation from Experiment 1 versus stimuli where the relation was reversed (right, red).

**FIGURE 4:**
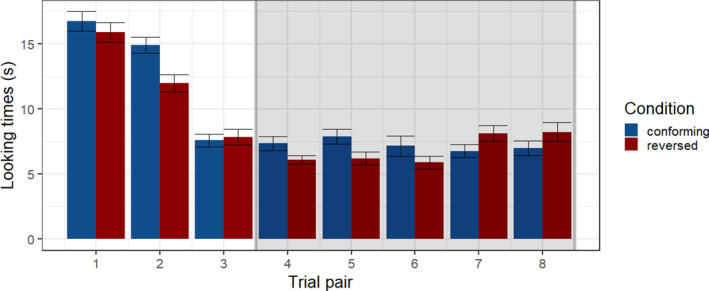
Barplots showing looking times (s, *y*‐axis) for individual trial pairs (*x*‐axis) for stimuli where the VOT‐F1 relation conformed to the trading relation from Experiment 1 versus a reversed relation. Overlayed in gray are the results of a Johnson‐Neyman test illustrating non‐significant trial pairs.

The anonymized data are on OSF: DOI 10.17605/OSF.IO/C7W8Q (https://osf.io/C7W8Q). To establish looking preferences for stimuli conforming to the trading relation versus a reversed relation, we calculated the average looking times across infants in each condition. In the conforming condition, average looking times were 10.06 s (SD = 6.07) and 9.28 s (SD = 5.51) in the reversed condition, as illustrated in Figure 3, resulting in an overall difference of 0.78 s. A linear mixed‐effects model for the looking times was designed using lme4 in R (Bates et al., 2015) with fixed effects for the conforming versus reversed condition and trial order, and random intercepts per participant, as well as an interaction between cue relation and trial order (see Appendix B Table B1). Looking times were log‐transformed and residuals of the model were normally distributed. The model revealed an effect of condition (*p* = 0.018, *d* = 0.49) with significantly longer looking times in the conforming compared to the reversed condition, a significant effect of trial order (*p* < 0.001, *d* = 0.2) as well as an interaction of condition and trial order (*p* = 0.016, *d* = 0.1). The interaction indicates that the change in looking time over the course of the experiment differs across conditions. A post‐hoc Johnson‐Neyman procedure was used to investigate the interaction and confirmed an interval of significantly longer looking times for the first 6 trials in the conforming compared to the first 6 trials in the reversed condition. While the data suggest a reversal of the looking times in the last 2 trials, this difference was not significant. Figure 4 shows looking times for each pair of trials contrasting in the conforming and reversed conditions and illustrates the trend of decreasing looking times over the course of the stimulus stream presentation, with the pattern of looking times in the last trials reversing compared to the earlier trials.

Experiment 2 revealed a difference in looking times between stimuli that exhibit the trading relation between VOT and F1 onset frequency compared to stimuli with a reversed relation. This result indicates that the 6‐month‐olds preferred listening to the stimuli conforming to the cue trading relation over those that show a reversed relation. Given that the experiment did not contain any familiarization phase and that the difference emerges in the first trials of the experiment, the preference for conforming stimuli appears not to be induced by exposure to the stimuli during the experiment but by a perceptual bias that the infants bring to the lab.

The interaction of trial order and looking time difference in the conforming and reversed condition suggests that as the infants became more exposed to the stimuli, their preference for the stimuli conforming to the trading relation diminished and, by the last trials, looking times for the reversed condition become longer. Such a pattern of looking time reduction and reversal is not inconsistent with existing literature when considering that infants may find the configuration of cues conforming to the trading relation more familiar. Preference for familiar versus novel stimuli is known to be modulated by exposure duration, with novelty preferences becoming more likely with longer exposure in infants at 6 months or younger (Hunter & Ames, 1988; Roder et al., 2000; Rose et al., 1982). In the context of the current experiment, a novelty preference would correspond to a preference for listening to the reversed relation stimuli, as infants presumably have had little to no exposure to such stimuli prior to their participation in our experiment. The current experiment was relatively long compared to typical head‐turn procedures with infants between 4 and 12 months; it included warmup trials followed by 16 test trial presentations compared to 8–12 trials as in Hirsh‐Pasek et al. (1987), Höhle et al. (2009) and Kemler Nelson et al. (1995).

## 4 GENERAL DISCUSSION

Previous research indicates that infants are sensitive to subphonemic cue relations in sound discrimination at a young age (Eimas, 1985; Miller & Eimas, 1983). Yet, as far as we know, no work has examined infants' preference for stimuli that do or do not adhere to the cue trading relation as demonstrated in the adult experiment. The current study investigated the role of the cue trading relation between VOT and F1 onset frequency in the perception of the German voicing contrast in both adult and infant listeners. Our goals were to establish whether a cue trading relation between VOT and F1 onset frequency is present in the perception of German stops and, if so, to assess whether 6‐month‐old infants show a preference for listening to streams of words that exemplify this relation versus streams that display a reversed relation. Our results confirm that the cue trading relation exists in German and that infants display a preference for listening to syllables exhibiting this relation over a reversed relation.

Note that in both conditions tested in Experiment 2, the individual cues, VOT and F1, varied to an equal amount (see Figure 2). Only their relation was different across the two conditions. Hence, any preference for stimuli in either condition must derive from the nature of the cue relation encoded in the two different conditions and not from the extent of variability present in individual cues. Extent of variability has been implicated in prior studies as an important factor in word learning, as infants have been shown to learn minimal pair non‐words involving the voicing contrast more robustly when exposed to stimuli with more acoustic variability (Höhle et al., 2020, 2021; Rost & McMurray, 2010). One interpretation of this result has been that the crucial phonetic contrast becomes more apparent when listeners are exposed to acoustically more variable stimuli. This is because, it is proposed in that interpretation, learners home in on the right cues for the crucial contrast by tracking the extent of variability in different candidate cues (Galle et al., 2015; Rost & McMurray, 2010). In this argument, cues that do not vary or vary less are promoted as the basis for discrimination and cues that vary more are demoted, with ‘less variable cues being more relevant for word learning than variable cues’ (Galle et al., 2015, p. 68; see also Rost & McMurray, 2010).

An alternative proposal (Höhle et al., 2020) capitalizes on the multiplicity of acoustic cues signaling phonemic contrasts and the stability of relations among these cues. It is in these relations where Höhle et al. (2020) trace at least part of the source for the benefit of acoustic variability in learning minimal pairs. Specifically, in the perspective of this alternative proposal, what appears to be ‘noise’, when considering variability at the level of individual acoustic parameters, is in fact crucial to the detection of relational properties among cues. Variability is essential to the identification of such relational properties between cues. This is so because, to find a stable relation between two or more cues, these cues must be allowed to vary individually (and the greater the range of their individual variabilities, the more robust the evidence for the presence of a relation among them). Thus, whereas Rost and McMurray (2010) as well as Apfelbaum and McMurray (2011) emphasize how variability helps prune out irrelevant signal dimensions, Höhle et al. (2020) emphasize that variability in relevant dimensions is also beneficial because it helps highlight relational properties among cues. Our present results of infants exhibiting preferences for specific cue relations over others do not speak directly to word learning. Yet, our results show that young infants are sensitive to relations across acoustic cues, thereby providing evidence for the plausibility of the hypothesis in Höhle et al. (2020).

In the context of language acquisition, the trading relation between VOT and F1 onset frequency is particularly noteworthy as it appears rooted in general perceptual mechanisms that are not necessarily language‐ or experience‐related (Kluender & Lotto, 1994). Early work on this relation traced a potential source of its origin in articulation. Specifically, Summerfield and Haggard (1977) originally proposed that the existence of this relation can be traced to the acoustic consequences of articulatory changes in the consonant‐vowel (CV) transition of voiceless aspirated stops: as the oral occlusion of a stop consonant is released, the F1 transition, which accompanies the articulator movement, increases from a low onset frequency to its steady‐state value for the subsequent vowel. As the onset of voicing in voiceless aspirated stops is delayed, so is the onset of the resonances forming the rising F1 formant transition (Liberman et al., 1958; Summerfield & Haggard, 1977). By the time F1 becomes audible, the articulators have progressed farther in the transition between the consonant and the vowel, resulting in a higher F1 frequency at the onset of voicing (Reetz & Jongman, 2020).

While VOT and F1 can be related in the production of stops in languages such as English, an exclusively articulatory origin for the VOT‐F1 trading relation is not consistent with the finding that the same relation is attested in the perception of the voiced‐voiceless contrast in Spanish. Voiceless stops are not typically aspirated and thus the F1 cue in these stops is minimally informative due to the lack of delay in voicing onset after the stop's release (Benkí, 2005; see also Pind, 1999 for a link between F1 and pre‐aspiration). This suggests that the VOT‐F1 relation, robustly present in the perceptual judgments of Spanish listeners, is either acquired from different, yet unidentified, articulatory processes in Spanish, or that this relation is rooted in general auditory processes irrespective of language experience as argued by Benkí (2005, p. 247). An auditory basis for the VOT‐F1 trading relation is also consistent with findings from two‐tone non‐speech analogues in which the onset frequency of the lower frequency component has a similar effect on voicing classification as F1 (Pisoni, 1977). Further evidence for general auditory mechanisms underlying the VOT‐F1 trading relation stems from work with non‐human species: certain bird species can be trained to associate different keys with either voiced or voiceless stops, after which they are exposed to ambiguous stimuli to see how different cues influence their response. Experiments with Japanese quail reveal similarities to human listeners (Kluender, 1991; Kluender & Lotto, 1994), as the birds categorized stops with ambiguous VOT as voiceless more often for higher F1 frequency than for lower F1 frequency stimuli. This result was replicated with budgerigars with no prior exposure to human speech (Flaherty et al., 2017). The lack of language exposure in the participants of these studies suggests that experience may not be necessary as a basis for the VOT‐F1 trading relation in perception.

These findings align with those in the current experiment. Since the infants had no exposure to the stimuli prior to the experiment, any preference for the trading relation over a reversed relation must stem either from infants' previous experience with the phonetic expression of these stops in their language input or from differences in how these cue trading relations are processed auditorily. Based on the results of our study, we cannot resolve the issue of whether language exposure is needed to acquire the VOT‐F1 trading relation by humans or whether the relation is inherent to the perceptual system. Systematic developmental data tracking infants' responsiveness to trading relations over time are lacking. To determine whether the preference for the VOT‐F1 relation is inherent to the perceptual system or acquired in the first months after birth, further evidence is required with infants at younger ages, ideally newborns.

In sum, our findings show that 6‐month‐olds prefer stimuli that conform to the cue trading relation in the language they are learning over a different cue relation. This indicates a perceptual bias for certain relations in the phonetic expression of phonological categories during the crucial developmental period of early phoneme category formation. Consequently, these results add to the plausibility of the hypothesis that acoustic variability is beneficial for infants' minimal pair learning because variability highlights the stability of the relations between individually varying cues.

## AUTHOR CONTRIBUTIONS

**Marc Hullebus**: Conceptualization; data curation; formal analysis; investigation; methodology; visualization; writing—original draft; writing—review and editing. **Adamantios Gafos**: Conceptualization; funding acquisition; methodology; formal analysis; software; resources; supervision; writing—review and editing. **Natalie Boll‐Avetisyan**: Formal analysis; methodology; validation; writing—review and editing. **Alan Langus**: Conceptualization; formal analysis; methodology; validation; writing—review and editing. **Tom Fritzsche**: Data curation; methodology; project administration; visualization; writing—review and editing. **Barbara Höhle**: Conceptualization; funding acquisition; methodology; resources; supervision; writing—review and editing.

## CONFLICT OF INTEREST STATEMENT

The authors declare no conflicts of interest with regard to the funding source for this study.

## Data Availability

Data are on OSF: DOI 10.17605/OSF.IO/C7W8Q (https://osf.io/C7W8Q).

## Footnotes

^1^
The pattern of results is the same when (as per a reviewer's request) male participants were excluded from the analysis.

## APPENDIX A

### Experiment 1: Logistic mixed effects model summary

**TABLE A1:** Voiceless Responses ∼ scaled (VOT) * scaled(F1) + (1|Subject).

Groups	Name	Variance	Std. Dev.
Random effects			
Subject	Intercept	0.3675	0.6062
Residual		0.30924	0.5561
Number of observations	4410		
Number of subjects	18		

	Estimate	Std. Error	z‐value	*p*‐value
Fixed effects				
Intercept	−0.39065	0.15194	−2.571	<0.05*
VOT (scaled)	2.88126	0.08674	33.217	<0.001***
F1 (scaled)	0.56211	0.05085	11.054	<0.001***
VOT (scaled): F1 (scaled)	−0.61494	0.07757	−7.927	<0.001***

## APPENDIX B

### Experiment 2: Linear mixed effects model summary

**TABLE B1:** Log looking time ∼ condition * trial + VOT + (1|Subject).

Groups	Name	Variance	Std. Dev.
Random effects			
Subject	(Intercept)	2,263,554	1505
Residual		23,161,677	4813
Number of observations	735		
Number of subjects	24		

	Estimate	Std. Error	Degr. Freedom	t‐value	*p*‐value
Fixed effects					
Intercept	16,089.1	629.2	193.0	25.571	<0.001***
Reversed condition	−2487.8	736.8	714.8	−13.479	<0.001***
Trial	−1454.2	107.9	719.4	−3.376	<0.001***
lowVOT	−274.1	358.7	716.3	−0.764	0.445
Reversed condition: Trial	484.8	153.3	719.3	3.162	<0.001**
